# Ago HITS-CLIP expands microRNA-mRNA interactions in nucleus and cytoplasm of gastric cancer cells

**DOI:** 10.1186/s12885-018-5246-0

**Published:** 2019-01-08

**Authors:** Xinyi Zhang, Bo Shen, Yalei Cui

**Affiliations:** 1 College of Life and Environmental Sciences, Hangzhou Normal University, Hangzhou, 311121 China; 2 College of Animal Science and Veterinary Medicine, Henan Agricultural University, Zhengzhou, 450002 Henan China

**Keywords:** Ago HITS-CLIP, miRNA-RNA interaction, Nucleus and cytoplasm, Gastric cancer cell

## Abstract

**Background:**

Intensive investigations have identified a collection of microRNAs (miRNAs) and their functional machineries in cytoplasm. However, a comprehensive view of miRNAs and mRNAs in cytoplasm and nucleus has not been explored. This study aims to reveal the mechanisms of miRNA-RNA interactions in nucleus and cytoplasm.

**Methods:**

In this study, the miRNAs and their target mRNAs in the Argonaute2 (Ago2) complex of nucleus and cytoplasm of gastric cancer cells were characterized using high-throughput sequencing of RNAs isolated by crosslinking immunoprecipitation (HITS-CLIP). Then, the selected miRNAs were verified by Northern blot. The target mRNAs in the Argonaute2 (Ago2) complex of nucleus and cytoplasm of gastric cancer cells were analyzed through Gene ontology (GO) and Kyoto encyclopedia of genes and genomes (KEGG) analysis.

**Results:**

The results revealed that there were 243 miRNAs and 265 miRNAs in the Ago2 complexes of nucleus and cytoplasm, respectively. The majority of mature miRNAs existed in cytoplasm. The analysis of miRNA targetome from the Ago2 complexes indicated that a lot of mRNAs with high expression level existed in nucleus. The target genes of miRNAs in the Ago2 complexes of nucleus and cytoplasm played important roles in cell proliferation, cell differentiation, innate immune response and tumorigenesis.

**Conclusions:**

microRNA-mRNA interactions occur in nucleus and cytoplasm of gastric cancer cells.

Therefore, our study demonstrated that miRNA-mRNA interactions not only took place in cytoplasm but also in nucleus.

## Background

MicroRNA (miRNA) as one kind of non-coding small RNA (~ 22 nucleotide) plays an important role in the regulation of gene expression [1, 2]. After the primary miRNA (pri-miRNA) is transcribed by RNA Pol II, it needs a series of processing to form the mature miRNA [1, 2]. The Drosha protein (~ 160 kDa) cleaves pri-miRNAs to initiate miRNA processing and maturation, and forms ~ 65 nt (nucleotide) precursor miRNAs with hairpin structures (pre-miRNAs) [3]. Then, the Dicer protein can act on the pre-miRNA and cleave the end loop of the pre-miRNA to generate a double-stranded RNA with a length of approximately 22 base pairs (bp) [1, 2]. The double-stranded RNA duplex leads RNA-induced silencing complex (RISC), containing a member of the conserved Argonaute (Ago) protein family, to target sites of mRNAs, resulting in the destabilization of the mRNAs and/or inhibition of translation [4, 5]. Generally, miRNA function as gene regulator mainly by targeting 3’UTR of mRNAs through the “seed” sequence (nucleotides 2–7) of miRNA [4–6]. Intensive investigations during the past decades have established miRNAs as key regulators of various biological processes including organ development, cell differentiation, cell proliferation, virus-host interactions and tumorigenesis [1, 2, 7]. However, more and more studies demonstrated that the function of miRNA needs to be further elucidated. Some researches reported that mature miRNAs could also be detected in nucleus [8]. *Meister G* found that miR-21 existed in both cytoplasm and nucleus [9]. As reported, miR-25 and miR-92a show clear nuclear enrichment in rat primary cortical neurons by using a combination of microarray analysis and small RNA deep sequencing [10]. Some reports have pointed out the existence of active RISC in nucleus because of the findings of human Ago proteins and other RNAi factors (DICER1, TARBP2 and GW182) in nucleus of cells [11–14]. However, the miRNAs in nucleus have not been intensively explored.

Generally, the interaction between a miRNA and its target requires base pairing with only 6–8 nucleotides. Therefore, it is a major challenge to predict target mRNAs. Based on evolutionarily conserved seed sequence and free binding energy, computational predictions have greatly improved the potentials to find bona fide miRNA target sites [15]. Nonetheless, different prediction algorithms produce a large number of divergent results for one given miRNA [16, 17]. In addition, computer programs usually predict targets on a genome-wide scale irrespective of mRNA and miRNA that are specific to tissues or cell lines. Thus, it has become increasingly important to develop biochemical tools based on physical interactions between miRNAs and their specific target RNAs. As miRNA target sites are parts of RISC that contain, except for mRNAs, Argonaute (Ago) protein and guiding miRNAs, co-immunoprecipitation (Co-IP) of Ago protein together with associated target mRNAs is an effective tool for the identification or validation of miRNA target candidates [18, 19].

In order to explore the miRNA-mRNA interaction in nucleus and cytoplasm, the miRNAs and mRNAs in the Ago complexes of nucleus and cytoplasm of gastric cancer cells (MGC-803) were characterized using high-throughput sequencing of RNAs isolated by crosslinking immunoprecipitation (HITS-CLIP) in this study. The results revealed that there existed 243 miRNAs and 265 miRNAs in Ago2 complex of nucleus and cytoplasm, respectively. The targetome analysis indicated that these miRNAs could function in nucleus or cytoplasm.

## Methods

### Cell culture

Human gastric cancer cells (MGC-803) were cultured in RPIM-1640 Medium (Gibco, USA) supplemented with 10% fetal bovine serum (FBS) at 37 °C. The cell line was purchased from The Cell Bank of Type Culture Collection of Chinese Academy of Sciences, Shanghai, China in the year of 2014.

### Isolation of nucleus and cytoplasm

MGC-803 cells were cross-linked at 254 nm and then washed twice with ice-cold phosphate-buffered saline (PBS). Subsequently, the cells were warmed at room for 45 s and lysed by TM-2 buffer [10 mM Tris-HCl, 2 mM MgCl2, phenylmethanesulfonyl fluoride (PMSF), pH 7.4] at room temperature for 1 min. To isolate nuclei and cytoplasm of cells, the lysate was centrifugated at 1000×g at 4 °C for 6 min.

### Western blot analysis

8% SDS-PAGE was used to separate the protein samples. Then the protein samples were transferred to a nitrocellulose membrane (Bio-Rad, USA) at 80 V for 1.5 h. The membrane was blocked with 3% milk at room temperature for 2 h. Subsequently, the membrane was incubated with a primary antibody (anti-tubulin IgG, anti-Lamin A/C IgG or anti-Ago2 IgG) at 4 °C overnight. Tubulin and Lamin A/C antibodies were purchased from Abcam (USA). The anti-Ago2 IgG was prepared in our laboratory. After washes with TBST, the membrane was immersed in 2% milk containing AP-conjugated goat antimouse IgG (Sigma, USA) for 1 h. At last, NBT and BCIP solutions (BBI, Canada) were used to detect the protein.

### Co-immunoprecipitation (co-IP) of Ago2 complex

The lysis buffer (20 mM Tris-HCl, 1 mM EDTA, 150 mM NaCl, 1% Triton X-100, pH 7.5) was prepared to lyse the isolated nuclei and cytoplasm. After treatment with RNAsin (Promega, USA), the lysate was incubated with RQ1 DNAase (Promega) for 5 min at 37°. Subsequently the Ago2 complex was immunoprecipitated using the polyclonal antibody against Ago2 for 3 h at 4 °C and then incubated with protein A-Sepharose (Bio-Rad) for 3 h at 4 °C. After washes with lysis buffer, the Ago2 complex was collected.

### Sequencing and sequence analysis of RNAs of Ago2 complex

RNA was extracted from immunoprecipitated Ago2 complex and subjected to sequencing using GA-I genome analyzer (Illumina, San Diego, CA). To analyze small RNAs, the ACGT V3.1 program developed by LC Sciences (Houston, USA) was employed. Then the high-quality sequences were obtained by removing adaptor sequences, mRNA, rRNA, tRNA, snRNA, snoRNA and other noncoding RNA sequences available in Rfam (http://www.sanger.ac.uk/software/Rfam). The high quality sequences were then blasted with known human miRNAs in miRBase 19.0 to obtain miRNAs in this study. To analyze the longer RNAs, trinity software and the Illumina paired end method developed by LC Sciences (Houston, USA) were employed. Then the high-quality reads were aligned to human mRNA database (ftp://ftp.ensembl.org/pub/release-70/fasta/homo_sapiens/).

### Northern blot

Total RNAs were extracted from the Ago2 complex of nucleus or cytoplasm using a mirVanaPTMP miRNA isolation kit according to the manufacturer’s protocol (Ambion, USA) and then separated on a denaturing 15% polyacrylamide gel containing 8 M urea. After transferred to a Hybond-N+ membrane (Amersham Biosciences, Buckinghamshire, UK), the RNAs were cross-linked under ultraviolet. The membrane was pre-incubated with DIG Easy Hyb granules buffer (Roche, Basel, Switzerland) for 1 h at 42 °C and then the miRNAs were probed against with complementary DIG-labeled miRNA probe at 42 °C overnight. The RNAs were detected using the DIG High Prime DNA Labeling and Detection Starter Kit II (Roche, USA). NBT and BCIP solutions (BBI, Canada) were used to detect the RNA.

### Prediction of genes targeted by miRNAs

The transcriptome sequences of the Ago2 complexes of nucleus and cytoplasm served as a database for miRNA target prediction. MiRNA target genes were predicted by employing TargetScan 5.1 (http://www.targetscan.org) and miRanda (http://www.microrna.org/) as described in previous study [18].

### Gene ontology (GO) and Kyoto encyclopedia of genes and genomes (KEGG) analysis

The coding sequences of transcripts obtained from the Ago2 complexes were aligned with the protein sequences in the GO database with the blast E value <1e^− 5^ [20]. The best hit GO identities were described the properties of these genes and gene products. The coding sequences of transcripts were analyzed by KEGG Pathway (239 KEGG) to analyze the relationship between genes and enzyme annotations in KEGG, and the information mapped to pathway.

## Results

### Acquirement of Ago2 complexes from cytoplasm and nucleus of gastric cancer cells

**Fig. 1:**
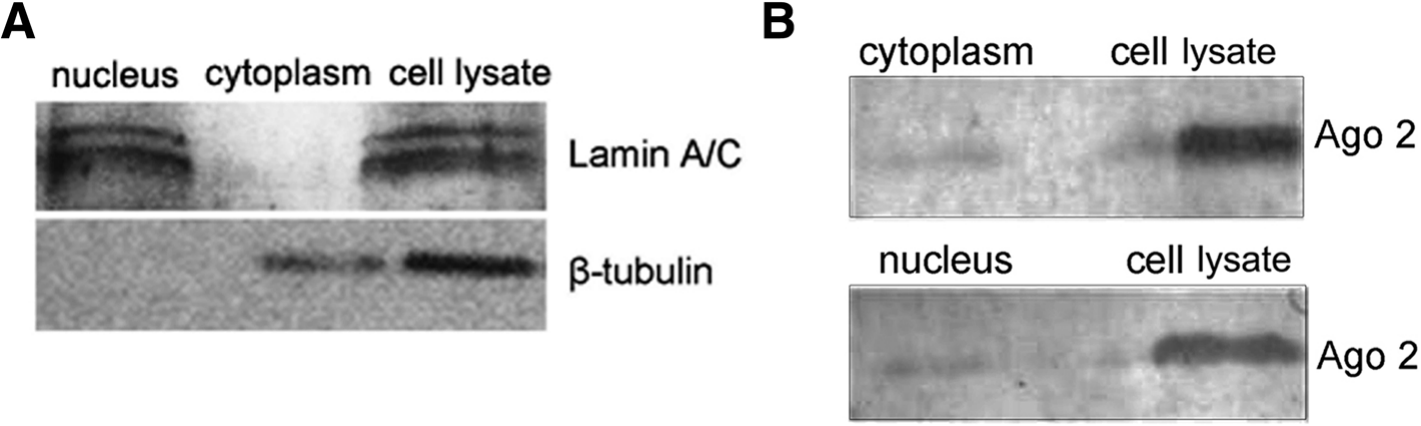
Acquirement of Ago2 complexes from cytoplasm and nucleus of gastric cancer cells. **a** Isolation of nuclei and cytoplasm. The nuclei and cytoplasm of gastric cancer cells (MGC-803) were isolated and then examined by Western blot analysis. Antibody against β-tubulin, a marker of cytoplasm, or Lamin A/C, a marker of nucleus was indicated on the right. **b** Co-immunoprecipitation of Ago2 complex of isolated nucleus and cytoplasm. Co-IP was conducted using isolated nucleus or cytoplasm with Ago2-specific antibody. The Co-IP products were detected by Western blot to detect the Ago2 protein

To identify miRNAs and mRNAs targeted by miRNAs in Ago2 complex of cytoplasm and nucleus, co-immunoprecipitation (Co-IP) assays with Ago2-specific antibody were performed using gastric cancer cells (MGC-803). The nuclear and cytoplasmic fractions of gastric cancer cells were extracted. Western blot analysis indicated that β-tubulin, a cytoplasmic marker, was detected in cell lysate and cytoplasm but not nucleus (Fig. 1a). At the same time, Lamin A/C, a nuclear marker, was found in cell lysate and nucleus (Fig. 1a). These data showed that cytoplasm and nucleus were successfully isolated.

The isolated cytoplasm and nucleus were subjected to Co-IP assays using Ago2-specific antibody. The results revealed that the Ago2 complexes of cytoplasm and nucleus were obtained (Fig. 1b).

### Characterization of miRNAs in Ago2 complexes of cytoplasm and nucleus of gastric cancer cells

**Fig. 2:**
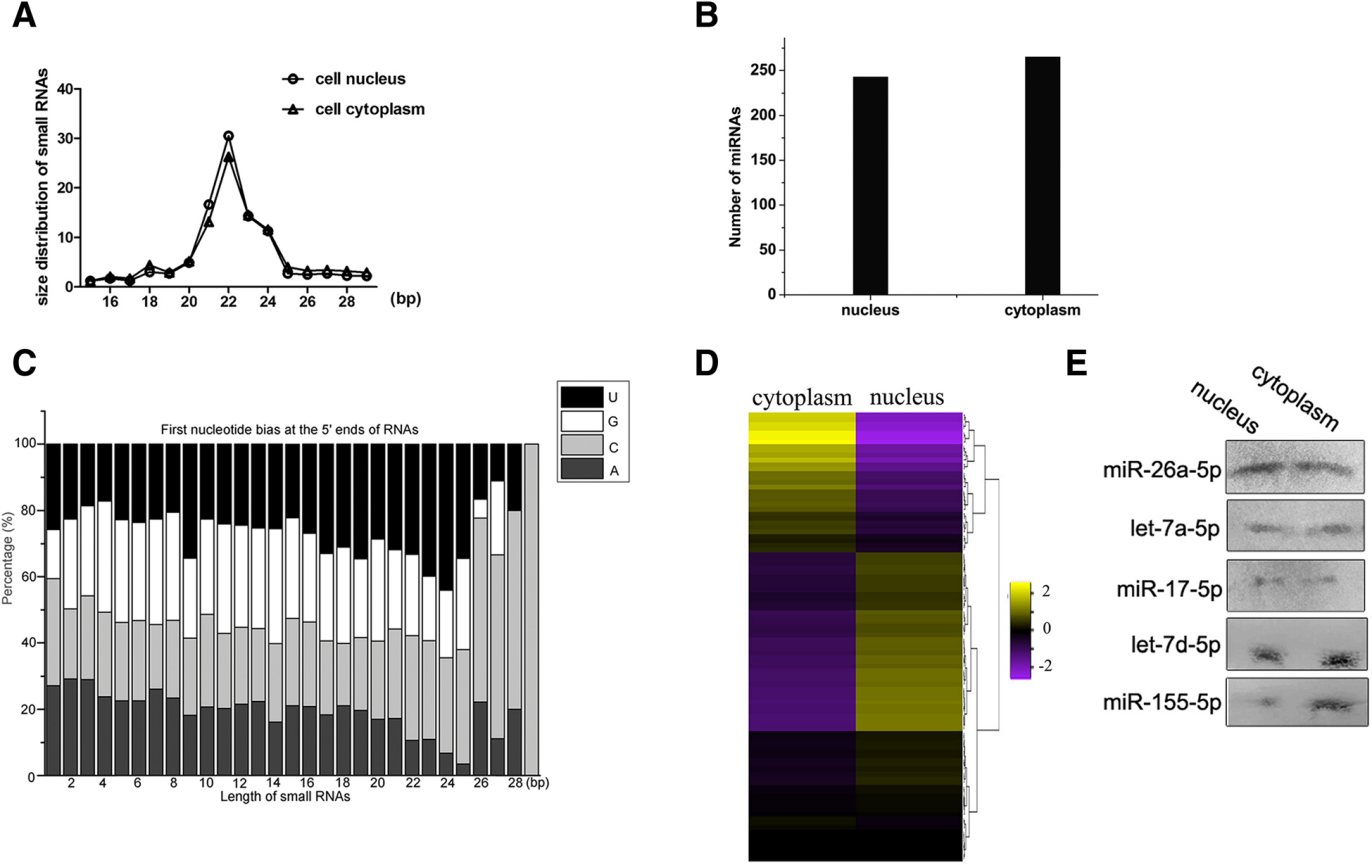
Characterization of miRNAs in Ago2 complexes of cytoplasm and nucleus of gastric cancer cells. **a** The distribution of small RNAs of different size in the Ago2 complex of nucleus and cytoplasm of gastric cancer cells. **b** The number of mature miRNAs in the Ago2 complex of nucleus and cytoplasm of gastric cancer cells. **c** First nucleotide preference of small RNAs from the Ago2 complex of gastric cancer cells. **d** Heat map of top 100 miRNAs with higher expression level in the Ago2 complex of nucleus and cytoplasm. The color indicated the Z-value of miRNAs. **e** Northern blot analysis of miRNAs. Five miRNAs, randomly selected from the Ago2 complex of nucleus and cytoplasm, were subjected to Northern blots. The probes used were indicated on the left

To characterize the miRNAs in Ago2 complexes of cytoplasm and nucleus, RNAs were extracted from Ago2 complexes and then subjected to deep sequencing. After removal of mRNA, rRNA, tRNA, snRNA and snoRNA sequences, high-throughput small RNA sequencing yielded an average of 7,813,944 high-quality reads. The results showed that most of small RNA reads were 20–25 nucleotides (nt) in length, which is typical for products generated by the enzyme Dicer (Fig. 2a). Of high-quality small RNA reads, 4,697,199 reads in nucleus and 4,545,958 in cytoplasm were mapped to known miRNAs in miRBase 19.0. In total, 243 miRNAs in the Ago2 complex of nucleus and 265 miRNAs in the Ago2 complex of cytoplasm were identified (Fig. 2b, Table 1), indicating that there was no difference of miRNA number between nucleus and cytoplasm of gastric cancer cells. The sequencing analysis revealed that there was a strong preference of U at the first nucleotide of small RNAs’ 5′ ends from the Ago2 complexes (Fig. 2c), showing that these small RNAs were in line with the characteristics of mature miRNA.

**Table 1:** MiRNAs in Ago2 complex of nucleus and cytoplasm of gastric cancer cells

miRNAs in Ago2 complex of nucleus			miRNAs in Ago2 complex of cytoplasm			
let-7a-3p	miR-18a-5p	miR-342-3p	let-7a-3p	miR-191-5p	miR-330-5p	miR-769-3p
let-7a-5p	miR-1908	miR-342-5p	let-7a-5p	miR-192-5p	miR-331-3p	miR-769-5p
let-7b-3p	miR-191-5p	miR-345-5p	let-7b-3p	miR-193a-3p	miR-331-5p	miR-873-5p
let-7b-5p	miR-192-5p	miR-34a-3p	let-7b-5p	miR-193a-5p	miR-335-3p	miR-876-5p
let-7c	miR-193b-3p	miR-361-3p	let-7c	miR-193b-3p	miR-335-5p	miR-877-5p
let-7d-3p	miR-194-5p	miR-361-5p	let-7d-3p	miR-193b-5p	miR-338-3p	miR-92a-5p
let-7d-5p	miR-196a-5p	miR-362-5p	let-7d-5p	miR-194-5p	miR-338-5p	miR-92a-3p
let-7e-5p	miR-196b-5p	miR-365b-3p	let-7e-5p	miR-196a-5p	miR-339-3p	miR-92b-3p
let-7f-1-3p	miR-197-3p	miR-374a-3p	let-7f-3p	miR-196b-3p	miR-339-5p	miR-92b-5p
let-7f-5p	miR-199a-5p	miR-374a-5p	let-7f-5p	miR-196b-5p	miR-33a-5p	miR-93-5p
let-7 g-5p	miR-199b-3p	miR-374b-5p	let-7 g-5p	miR-197-3p	miR-340-3p	miR-941
let-7i-5p	miR-19a-3p	miR-378a-3p	let-7i-5p	miR-199a-5p	miR-340-5p	miR-95
miR-100-3p	miR-19b-3p	miR-421	miR-100-3p	miR-199b-3p	miR-342-3p	miR-96-5p
miR-100-5p	miR-200c-3p	miR-423-3p	miR-100-5p	miR-199b-5p	miR-342-5p	miR-98-3p
miR-101-3p	miR-203a	miR-423-5p	miR-101-3p	miR-19a-3p	miR-345-5p	miR-98-5p
miR-103a-3p	miR-20a-5p	miR-424-3p	miR-101-5p	miR-19b-3p	miR-34a-3p	miR-99a-3p
miR-105-5p	miR-210	miR-424-5p	miR-103a-3p	miR-200c-3p	miR-34a-5p	miR-99a-5p
miR-106b-3p	miR-2110	miR-425-3p	miR-106b-5p	miR-203a	miR-361-3p	miR-99b-3p
miR-106b-5p	miR-212-5p	miR-425-5p	miR-107	miR-20a-5p	miR-361-5p	miR-99b-5p
miR-107	miR-21-3p	miR-4454	miR-10a-3p	miR-210	miR-362-5p	
miR-10a-3p	miR-215	miR-450a-5p	miR-10a-5p	miR-2110	miR-365b-3p	
miR-10a-5p	miR-21-5p	miR-450b-5p	miR-10b-5p	miR-212-5p	miR-3687	
miR-10b-5p	miR-221-3p	miR-452-5p	miR-1180	miR-21-3p	miR-374a-3p	
miR-1180	miR-221-5p	miR-454-3p	miR-1247-3p	miR-214-3p	miR-374a-5p	
miR-1247-5p	miR-222-3p	miR-454-5p	miR-1255a	miR-21-5p	miR-374b-3p	
miR-1254	miR-22-3p	miR-455-3p	miR-125a-3p	miR-219-5p	miR-374b-5p	
miR-1255a	miR-224-3p	miR-4677-3p	miR-125a-5p	miR-221-3p	miR-378a-3p	
miR-125a-3p	miR-224-5p	miR-484	miR-125b-3p	miR-221-5p	miR-421	
miR-125a-5p	miR-23a-3p	miR-500a-3p	miR-125b-5p	miR-222-3p	miR-423-3p	
miR-125b-2-3p	miR-23b-3p	miR-501-3p	miR-126-3p	miR-22-3p	miR-423-5p	
miR-125b-5p	miR-24-2-5p	miR-502-3p	miR-126-5p	miR-224-3p	miR-424-3p	
miR-126-3p	miR-24-3p	miR-503-5p	miR-1268a	miR-224-5p	miR-424-5p	
miR-126-5p	miR-25-3p	miR-505-5p	miR-1277-3p	miR-22-5p	miR-425-3p	
miR-1277-3p	miR-25-5p	miR-532-3p	miR-1277-5p	miR-2355-5p	miR-425-5p	
miR-128	miR-26a-5p	miR-532-5p	miR-128	miR-23a-3p	miR-450b-5p	
miR-1285-3p	miR-26b-3p	miR-545-3p	miR-1285-3p	miR-23b-3p	miR-452-5p	
miR-1285-5p	miR-26b-5p	miR-548a-3p	miR-1301	miR-24-5p	miR-454-3p	
miR-1291	miR-27a-3p	miR-548d-5p	miR-1303	miR-24-3p	miR-454-5p	
miR-1292-5p	miR-27b-3p	miR-548e	miR-1304-3p	miR-25-3p	miR-455-3p	
miR-1301	miR-27b-5p	miR-550a-5p	miR-1307-3p	miR-25-5p	miR-4677-3p	
miR-1303	miR-28-3p	miR-570-3p	miR-130a-3p	miR-26a-5p	miR-484	
miR-1304-3p	miR-28-5p	miR-574-3p	miR-130b-3p	miR-26b-3p	miR-491-5p	
miR-1307-3p	miR-296-3p	miR-574-5p	miR-130b-5p	miR-26b-5p	miR-500a-3p	
miR-130a-3p	miR-29a-3p	miR-576-3p	miR-132-3p	miR-27a-3p	miR-501-3p	
miR-130b-3p	miR-29b-3p	miR-582-3p	miR-132-5p	miR-27a-5p	miR-502-3p	
miR-130b-5p	miR-29c-3p	miR-582-5p	miR-140-3p	miR-27b-3p	miR-503-5p	
miR-132-3p	miR-301a-3p	miR-589-5p	miR-141-3p	miR-27b-5p	miR-505-3p	
miR-132-5p	miR-301a-5p	miR-590-3p	miR-143-3p	miR-28-3p	miR-505-5p	
miR-1343	miR-301b	miR-615-3p	miR-145-5p	miR-28-5p	miR-516a-5p	
miR-135b-5p	miR-3065-5p	miR-629-5p	miR-146a-5p	miR-296-3p	miR-532-3p	
miR-140-3p	miR-3074-5p	miR-641	miR-146b-5p	miR-296-5p	miR-532-5p	
miR-143-3p	miR-30a-3p	miR-652-3p	miR-148a-3p	miR-29a-3p	miR-545-3p	
miR-146a-5p	miR-30a-5p	miR-660-5p	miR-148a-5p	miR-29b-3p	miR-548a-3p	
miR-146b-5p	miR-30b-3p	miR-671-3p	miR-148b-3p	miR-29c-3p	miR-548d-5p	
miR-148a-3p	miR-30b-5p	miR-675-3p	miR-148b-5p	miR-301a-3p	miR-548e	
miR-148a-5p	miR-30c-2-3p	miR-7-1-3p	miR-149-5p	miR-301a-5p	miR-550a-5p	
miR-148b-3p	miR-30c-5p	miR-744-5p	miR-151a-3p	miR-301b	miR-556-5p	
miR-148b-5p	miR-30d-3p	miR-7-5p	miR-151a-5p	miR-3074-5p	miR-561-5p	
miR-149-5p	miR-30d-5p	miR-766-3p	miR-151b	miR-30a-3p	miR-570-3p	
miR-151a-3p	miR-30e-3p	miR-767-5p	miR-152	miR-30a-5p	miR-574-3p	
miR-151a-5p	miR-30e-5p	miR-769-5p	miR-155-5p	miR-30b-3p	miR-574-5p	
miR-151b	miR-31-3p	miR-873-3p	miR-15a-5p	miR-30b-5p	miR-576-3p	
miR-152	miR-31-5p	miR-873-5p	miR-15b-3p	miR-30c-3p	miR-582-3p	
miR-155-5p	miR-3168	miR-876-5p	miR-15b-5p	miR-30c-5p	miR-582-5p	
miR-15a-5p	miR-320a	miR-877-5p	miR-16-2-3p	miR-30d-3p	miR-589-5p	
miR-15b-3p	miR-320b	miR-92a-1-5p	miR-16-5p	miR-30d-5p	miR-590-3p	
miR-15b-5p	miR-320c	miR-92a-3p	miR-17-3p	miR-30e-3p	miR-615-5p	
miR-16-2-3p	miR-320d	miR-92b-3p	miR-17-5p	miR-30e-5p	miR-625-5p	
miR-16-5p	miR-324-3p	miR-92b-5p	miR-181a-3p	miR-31-3p	miR-628-5p	
miR-17-3p	miR-324-5p	miR-93-3p	miR-181a-5p	miR-31-5p	miR-629-5p	
miR-17-5p	miR-32-5p	miR-93-5p	miR-181b-5p	miR-3168	miR-641	
miR-181a-2-3p	miR-330-3p	miR-941	miR-181c-3p	miR-3196	miR-652-3p	
miR-181a-5p	miR-330-5p	miR-95	miR-181c-5p	miR-320a	miR-660-5p	
miR-181b-5p	miR-331-3p	miR-96-5p	miR-181d	miR-320b-3p	miR-664a-3p	
miR-181c-3p	miR-335-3p	miR-98-3p	miR-182-5p	miR-320b-5p	miR-671-3p	
miR-181c-5p	miR-335-5p	miR-98-5p	miR-183-3p	miR-320c	miR-675-3p	
miR-181d	miR-338-3p	miR-99a-3p	miR-183-5p	miR-320d	miR-675-5p	
miR-182-5p	miR-338-5p	miR-99a-5p	miR-185-5p	miR-32-3p	miR-7-3p	
miR-183-3p	miR-339-3p	miR-99b-5p	miR-186-3p	miR-324-5p	miR-744-5p	
miR-183-5p	miR-33a-5p		miR-186-5p	miR-32-5p	miR-7-5p	
miR-185-5p	miR-340-3p		miR-18a-5p	miR-326	miR-766-3p	
miR-186-5p	miR-340-5p		miR-1908	miR-330-3p	miR-767-5p	

The results indicated that the expression profile of miRNAs in cytoplasm was different from that in nucleus (Fig. 2d). A lot of mature miRNAs existed in cytoplasm (Fig. 2d).

To confirm the existence of mature miRNAs in the Ago2 complexes of cytoplasm and nucleus, 5 miRNAs were randomly selected for Northern blot analysis. The results of Northern blots were consistent with those of miRNA sequencing (Fig. 2e).

### Identification of miRNA targetome in Ago2 complexes of nucleus and cytoplasm of gastric cancer cells

**Fig. 3:**
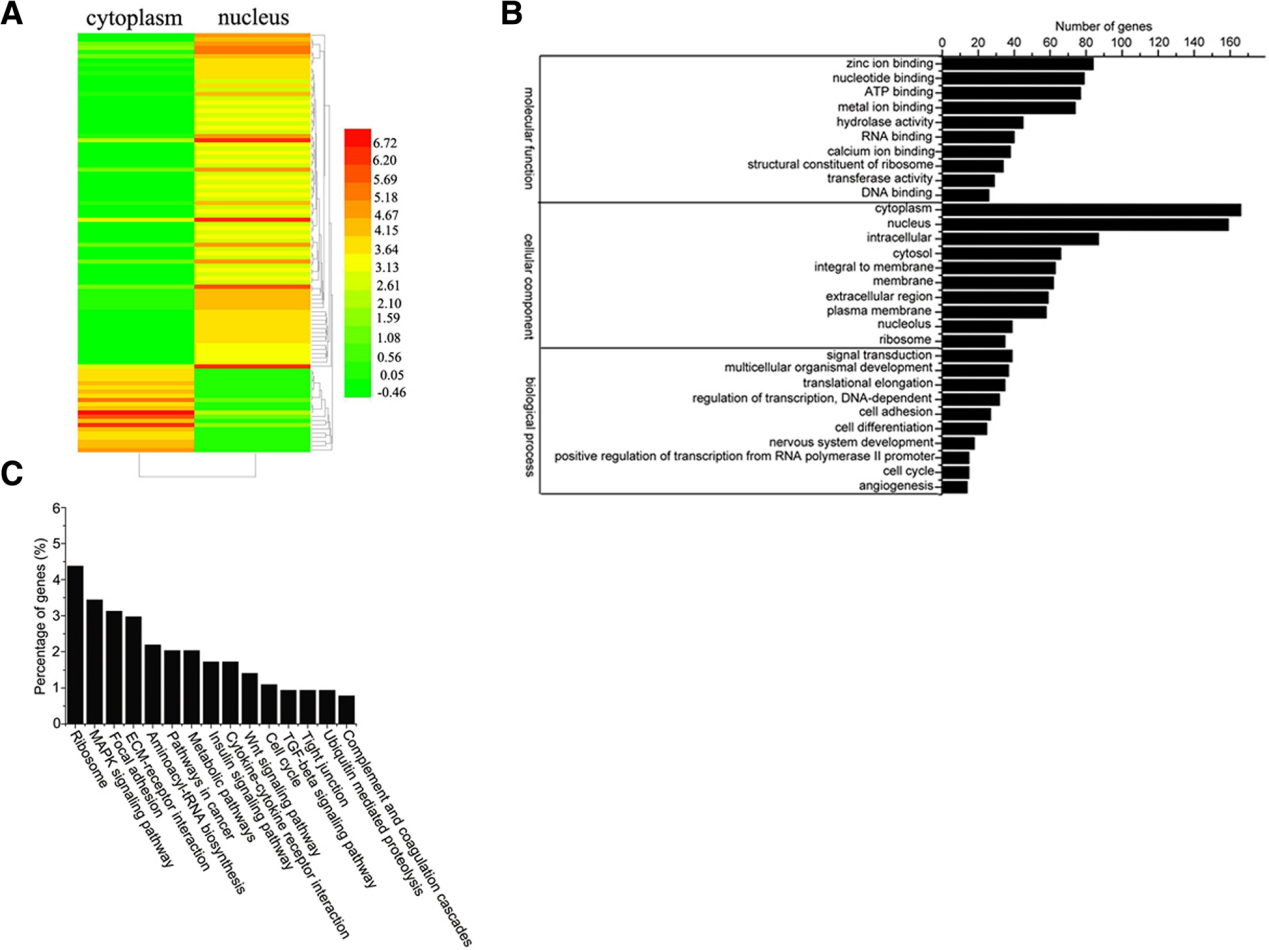
Identification of miRNA targetome in Ago2 complexes of nucleus and cytoplasm of gastric cancer cells. **a** Heat map of differentially expressed top 100 mRNAs in the Ago2 complexes of nucleus and cytoplasm of gastric cancer cells. The number represented the expression level of genes. **b** GO analysis of differentially expressed genes in the Ago2 complexes of nucleus and cytoplasm. **c** KEGG classifications of differentially expressed genes in the Ago2 complexes of nucleus and cytoplasm

To obtain an overview of gene expression profiles of miRNA targetome in cytoplasm and nucleus, cDNA libraries of target mRNA tags of miRNAs were prepared from the Ago2 complexes of cytoplasm and nucleus. After removal of repetitive and low-quality reads, an average of 12.2 million high-quality reads were aligned to human mRNA database. A total of 191,134 unigenes in nucleus and 191,134 unigenes in cytoplasm were obtained. The results showed that a large proportion of the target mRNA reads, 64.7% in nucleus and 55% in cytoplasm, were mapped to human mRNA database. Among the top 100 mRNAs with higher expression level, 80 genes were downregulated in cytoplasm and 20 upregulated (Fig. 3a), indicating that the main proportion of mRNAs existed in nucleus.

The analysis of cellular component by gene ontology (GO) revealed that the differentially expressed genes could be categorized into molecular function, cellular component and biological process (Fig. 3b). In the molecular function category, the most common functional annotations were the zinc ion binding, nucleotide binding, ATP binding and metal ion binding (Fig. 3b). A lot of differentially expressed genes could function as cellular components of cytoplasm and nucleus (Fig. 3b). In terms of biological process, most of genes were implicated in signal transduction, multicellular organismal development and translational elongation (Fig. 3b). The results of KEGG analysis indicated that the differentially expressed genes were classified into 239 KEGG pathways. Many genes were involved in the MAPK signaling pathway, the Wnt signaling pathway, focal adhesion, the TGF-beta signaling pathway and pathways in cancer (Fig. 3c). These data showed that the target genes of miRNAs in the Ago2 complexes of nucleus and cytoplasm played important roles in cell proliferation, cell differentiation, innate immune response and tumorigenesis.

### Pathways mediated by miRNAs with higher expression level

**Fig. 4:**
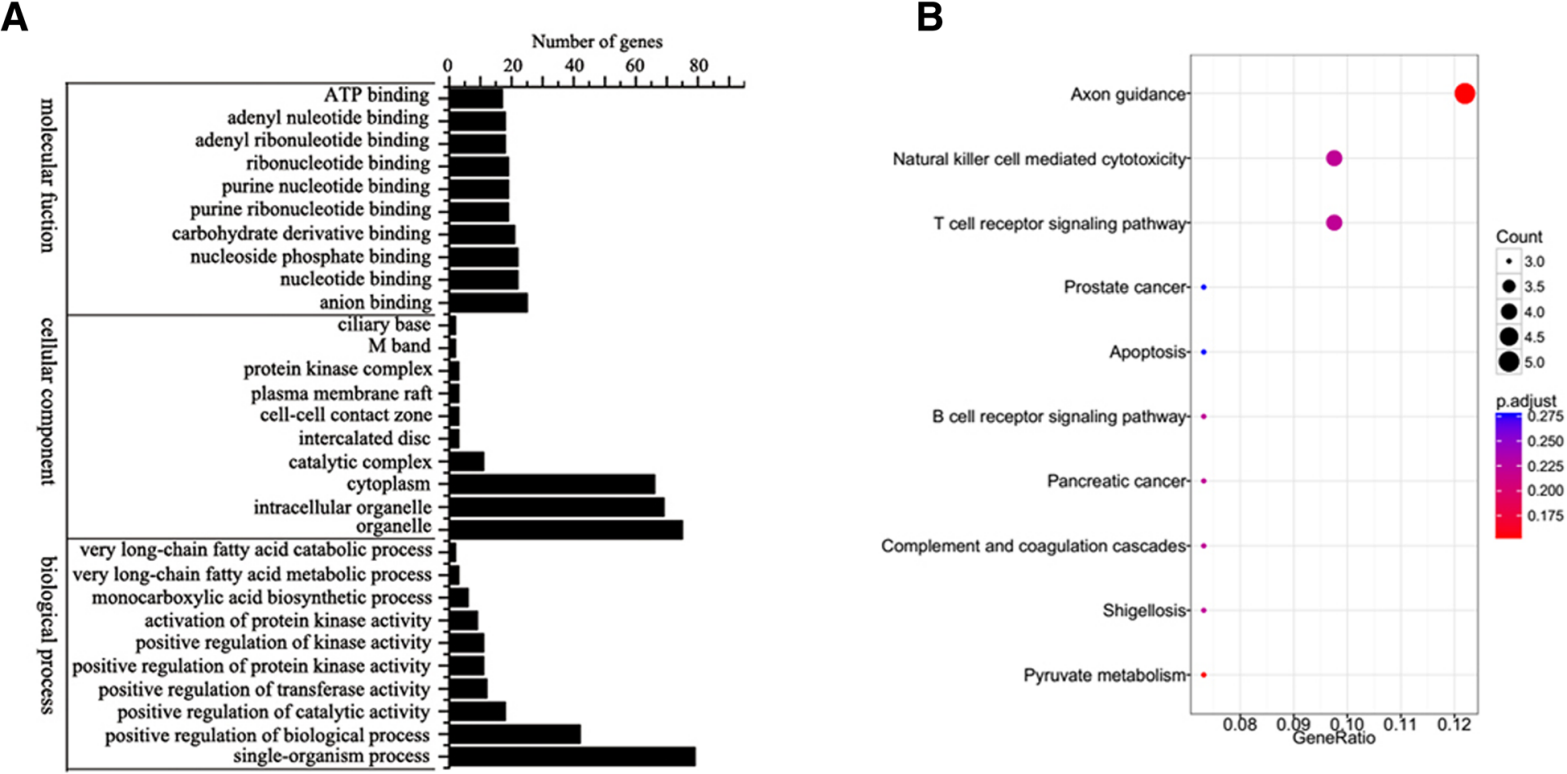
Pathways mediated by miRNAs with higher expression level. **a** GO analysis of transcripts identified as targets of 10 miRNAs with higher expression level in cytoplasm and nucleus. **b** KEGG classifications of genes targeted by 10 miRNAs with higher expression level in cytoplasm and nucleus

In order to characterize the potential interactions between miRNAs and their targets in cytoplasm and nucleus, 10 miRNAs with higher expression level in nucleus and cytoplasm (miR-125b-5p, miR-92b-3p, miR-17-5p, miR-30b-5p, miR-20a-5p, miR-21-3p, let-7d-3p, miR-98-5p, miR-424-5p and miR-155-5p) and their predicted targets were subjected to GO and KEGG analyses. The results of GO analysis demonstrated that most of the miRNA targets were involved in single-organism process, positive regulation of biological activity and cellular component including cytoplasm, intracellular organelle and organelle (Fig. 4a). The data of KEGG analysis revealed that most target genes participated in axon guidance, natural killer cell mediated cytotoxity and T cell receptor signaling pathway.

## Discussion

Over a decade of years, investigations have identified a collection of miRNA functional machineries. Recently, more and more investigations focus on miRNA functions in nucleus. It is found that miR-9 can result in degradation of MALAT-1 mRNA by targeting the metastasis-associated lung adenocarcinoma transcript 1 (MALAT-1) in nucleus [21]. MiR-671 could regulate the expression level of the circular non-coding (ncRNA) natural antisense transcript in nucleus [22]. These studies demonstrate that miRNAs in nucleus can also regulate the expressions of genes. Consistent with previous studies, our study characterized 243 miRNAs and 265 miRNAs in the Ago2 complexes of nucleus and cytoplasm of gastric cancer cells in this study. Report has shown that miRNA could translocate from cytoplasm to nucleus, which has been demonstrated by using superquencher molecular probes complementary to the mature miR-122 [23]. And RISC-associated proteins are involved in transport from the cytoplasm to the nucleus in human cells [24, 25]. For instance, Exportin-1 and importin-8 play positive roles in the nuclear import of miRNAs [24, 25]. However, miRNAs in nucleus and cytoplasm have not been extensively compared. In the present study, the results revealed that the expression pattern of miRNAs in cytoplasm of gastric cancer cells were obviously different from that in nucleus. The majority of mature miRNAs existed in cytoplasm. Therefore, our study contributed a comprehensive view of miRNAs and their targets in nucleus and cytoplasm.

MiRNAs, loaded into the miRISC, are thought to target multiple mRNAs, affecting the translation or stability of several target mRNAs [2, 4, 6, 26]. In this study, the miRNA-mRNA map in nucleus and cytoplasm was investigated using high-throughput sequencing of RNAs isolated by crosslinking immunoprecipitation (HITS-CLIP). The targetome of Ago2 complexes in nucleus and cytoplasm was obtained. The results showed that the main proportion of mRNAs existed in nucleus, while miRNAs function predominantly in cytoplasm. The existence of miRNAs in the Ago2 complexes of nucleus and cytoplasm revealed that miRNAs could function in nucleus and cytoplasm. Decades of studies have elucidated the function and mechanism of miRNAs in cytoplasm [1, 2, 4, 5]. In recent years, more and more investigations focus on the function and mechanism of miRNAs in nucleus. In *C. elegans*, it is reported that the intronic regions of pre-mRNA could be silenced by dsRNAs [27]. In human HeLa cells, the U6 small nuclear RNA transcripts are also susceptible to RNAi-mediated post-transcriptional silencing [28]. These findings support our miRNA-mRNA-Ago interactions in nucleus of gastric cancer cells. In nucleus, miR-320 can trigger the heterochromatinization and transcriptional gene silencing of POLR3D by binding the promoter locus of POLR3D [29]. In addition, some splicing factors, such as U2 and U5 snRNP core subunits, SRSF1, SRSF3, SRSF7, SRSF10, PTBP1, PTBP2, KHDRBS1 and snRNAs, could be detected in immunoprecipitated Ago complex, indicating a cross-talk between splicing factors and nuclear RISC [30]. However, the regulatory mechanism of nuclear miRNAs needs to be further investigated.
